# ECM and epithelial stem cells: the scaffold of destiny

**DOI:** 10.3389/fcell.2024.1359585

**Published:** 2024-03-20

**Authors:** Soline Estrach, Charles-Maxime Vivier, Chloé C. Féral

**Affiliations:** INSERM, CNRS, IRCAN, Université Côte d’Azur, Nice, France

**Keywords:** adult epithelial stem cells, ECM, mechanosignaling, aging, stemness

## Abstract

Adult stem cells play a critical role in maintaining tissue homeostasis and promoting longevity. The intricate organization and presence of common markers among adult epithelial stem cells in the intestine, lung, and skin serve as hallmarks of these cells. The specific location pattern of these cells within their respective organs highlights the significance of the niche in which they reside. The extracellular matrix (ECM) not only provides physical support but also acts as a reservoir for various biochemical and biophysical signals. We will consider differences in proliferation, repair, and regenerative capacities of the three epithelia and review how environmental cues emerging from the niche regulate cell fate. These cues are transduced via mechanosignaling, regulating gene expression, and bring us to the concept of the fate scaffold. Understanding both the analogies and discrepancies in the mechanisms that govern stem cell fate in various organs can offer valuable insights for rejuvenation therapy and tissue engineering.

## Introduction

The behaviour of nearly all stem cells, regardless of pluripotent or tissue-specific, embryonic or adult, is driven and regulated by an intricated regulatory pathway of intrinsic transcriptional programs and extrinsic signals (Watt and Driskell, 2010). These extrinsic signals predominantly originate from the local microenvironment or niche. It is becoming increasingly clear that the extracellular matrix (ECM) is a crucial component for stem cells, and biophysical properties regulate fate decisions over time. For example, it has been shown in liver regeneration that the replacement of depleted hepatic cells can occur partly through the proliferation of some mature adult hepatocytes and other hepatic cell types (Li et al., 2019). In this review, we are interested in organs which display renewal by specific subpopulations of stem cells. We will describe the different epithelial stem populations, focusing on the main epithelia that display a differential proliferation rate, namely the Gut, Lung, and Skin. Subsequently, we will highlight the specialized ECM components that compose the niche in each model and finally expose the mechanotransduction signaling effects on stem cell fate in each organ. We will shed light on the common mechanisms used by niche biophysical attributes to regulate cell fate in all adult epithelia and emphasize this growing field in the perspective of rejuvenating research.

## Adult epithelial stem cells

### Intestinal stem cells

Adult epithelial stem cells in the intestine play a critical function in sustaining the regenerative ability of this dynamic organ. The intestine is known as one of the most renewing organ in the body, replacing its surface every five to 7 days (Meran et al., 2017). This continuous cell renewal is essential for the epithelium to withstand the constant challenges it faces during the absorption of nutriments, and evacuation of waste material.

**FIGURE 1:**
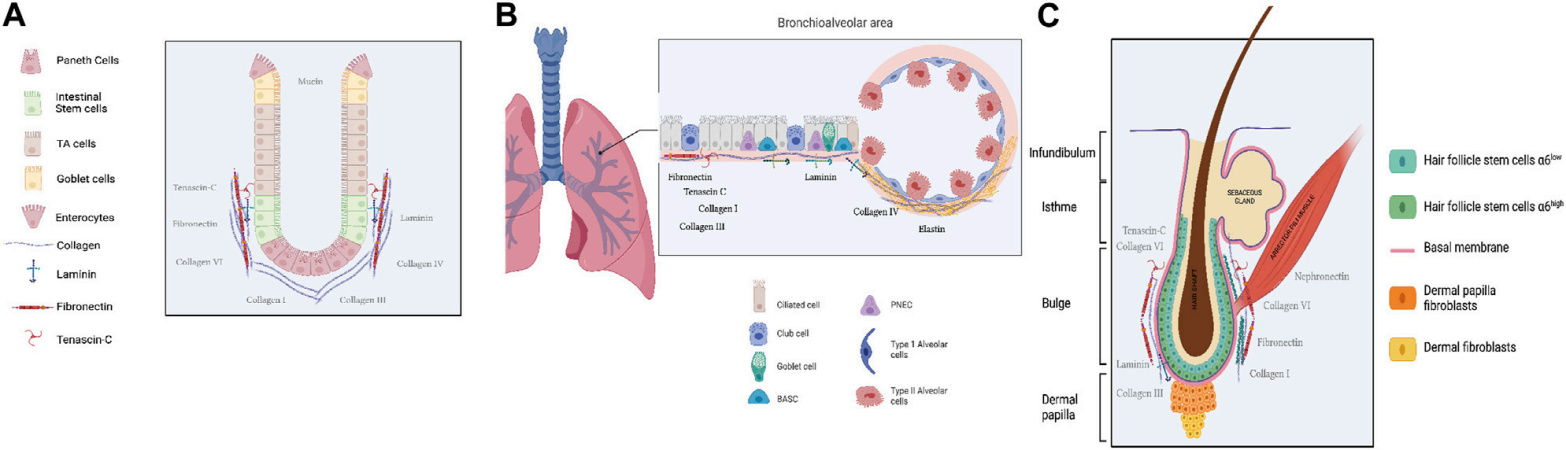
Schematic representation of adult stem cell location in the three epithelia Gut **(A)**, Lung **(B)** and Skin **(C)**. Created with Biorender. com, accession 21 December 2023.

The intestinal epithelium is composed of two independent compartments: the proliferative crypts of Lieberkühn and the long finger-like structures called villi. Those structures are no longer able to divide (Figure 1A). Intestinal stem cells (ISCs) belong to the intestinal crypts, and they constantly divide to maintain the high level of renewal of the intestinal epithelium. ISCs give rise to either another stem cell for self-renewal or a progenitor cell that rapidly divides before undergoing terminal differentiation (Baulies et al., 2020).

Early studies suggested the existence of two independent ISC populations: the crypt base columnar (CBC) cells and the +4 cells. Recent discoveries in transcriptomics and lineage-tracing tools have led to the identification of numerous putative stem cell-specific genes marking the CBC cells or +4 cells, leading to direct tracing of their attached progenies. A landmark study in 2007 identified leucine-rich repeat-containing G protein-coupled receptor 5 (Lgr5) as a genuine ISC marker specific to CBC cells (Barker et al., 2007). Subsequent studies proposed alternative ISC markers predominantly enriched at the +4 cells, including polycomb complex protein Bmi1 (Sangiorgi and Capecchi, 2008), homeodomain-only protein (Hopx) (Takeda et al., 2011), and mouse telomerase reverse transcriptase (Tert) (Montgomery et al., 2011).

In homeostasis, Lgr5+ cells strategy is better for long-term lineage-tracing than using +4 markers (Barker et al., 2007). Moreover, the expression of leucine-rich repeats and immunoglobulin-like domains 1 (Lrig1) has been identified in both CBC cells and +4 ISCs (Powell et al., 2012; Wong et al., 2012). Powell et al. suggested Lrig1 as a marker of a distinct class of label-retaining and slow-cycling cells in the lower crypts.

### Lung stem cells

The adult lung is a complex organ consisting of a branched airway network that includes the trachea, bronchi, bronchioles, and alveoli (Figure 1B). In mice the alveolar compartment, where the airways open at the bronchioalveolar duct junctions (BADJs), stays largely quiescent in the uninjured lung and most cells in this niche have a moderate turnover. However, the lung displays outstanding repair capacity under injury, with renewal turnover occurring over several years. Recent single-cell studies have revealed significant functional heterogeneity in the respiratory system, emphasizing its highly adaptive properties (Basil et al., 2020).

To regenerate proper functions, the lung requires distinct progenitor cell populations, and various small populations of progenitor cells have been identified in the regeneration of the distal airway epithelium after injury in mice (Perl et al., 2005; Bertoncello and McQualter, 2010; Chen et al., 2012; Barkauskas et al., 2013). A population meeting those criteria is the variant of club/secretory cells (V-club cells), identified by their location near neuroendocrine bodies and low expression of cytochrome Cyp2f2. Upk3a is another marker of the V-club cell population that can differentiate into club cells and ciliated cells during homeostasis or after naphthalene airway injury (Giangreco et al., 2008; T et al., 2011).

Few studies describe additional potential for the V-club/secretory cells that can differentiate into AT2 cells during bleomycin-induced injury, revealing their ability to be mobilized and to migrate more distally in the lung (Zheng et al., 2017). In addition to AT2 cells, supplemental progenitor cells participate in alveolar repair after injury in murine model. AT2 cells in adult can display both self-renewing stem cell-like properties and regenerating capacity after injury (Barkauskas et al., 2013). Axin2, a transcriptional target of Wnt signaling, is expressed in a sub part of the AT2 population displays a predominant role during repair of the lung alveolus after acute injury (Nabhan et al., 2018; Zacharias et al., 2018).

Another discrete population progenitor cells have been characterized at the branching point between distal murine airways and alveoli, named bronchoalveolar stem cells (BASCs). BASCs are defined by the expression of the secretory cell marker Scgb1a, the AT2 marker Sftpc (Kim et al., 2005), and the stem cell antigen-1 (Sca1) (Raiser and Kim, 2009). BASCs are able to self-renew and display multipotent differentiation features into Club, AT1, and AT2 cells as documented by genetic tracing studies (Liu et al., 2019; Salwig et al., 2019).

### Skin stem cells

The skin serves as the initial and extensive barrier organ in the mammalian body. It has a resilient and compliant organization that gives way to adaption to external conditions by quickly fixing mechanical, chemical, and biological injuries (Watt, 2014). The skin is composed of different layers, from the deepest to the most external (Figure 1C): **Hypodermis:** This is a subcutaneous layer of fat that supplies nutrients to the upper layers, cushions, and insulates the body. It is located beneath the dermis. **Dermis:** The dermis acts as a scaffold for the epidermis. It is a fibrous layer primarily composed of extracellular matrix (ECM) but also contains several cell types, including fibroblasts and immune cells. **Epidermis:** The epidermis is the outermost layer and forms the protective structure of the skin. It is composed of a stratified epithelium, called the interfollicular epidermis, covered by a layer of cornified dead cells that protect the entire epithelium, along with associated appendages such as hair follicles and sebaceous glands. The maintenance of the epidermis throughout life involves the proliferation of stem cells and the differentiation of their progeny. Various epithelial stem cell (SC) populations contribute to skin homeostasis, and among them, the most characterized is the Hair Follicle Stem Cells (HFSCs) which is located in the permanent portion of the hair follicle, ranging from the bulge to the junctional zone (Schepeler et al., 2014).

In undamaged skin, different cells colonize distinct and limited areas in the hair follicle. However, after tissue injury, these cells exhibit the remarkable ability to give rise to all epidermal cells, including the interfollicular epidermis, which is situated between the hair follicles and comprises the largest pool of keratinocytes in the skin. Hair follicle stem cells (HFSCs) are characterized by the expression of several markers such as integrin α6, CD34, keratins (K) K15, K19, LIM homeobox 2 (Lhx2), SOX9, leucine-rich repeat-containing G protein-coupled receptor 5 (Lgr5), leucine-rich repeats and immunoglobulin-like domains 1 (Lrig1), and Col17A1 (Morris et al., 2004; Tumbar et al., 2004; Rhee et al., 2006; Jaks et al., 2008; Nowak et al., 2008; Jensen et al., 2009; Matsumura et al., 2016).

Additionally, leucine-rich repeats and immunoglobulin-like domains 1 (Lrig1) positive cells, are located in the junctional zone in the upper isthmus and contribute to all three skin epithelial lineages in grafting experiments. During growth, bulge cells give rise to progeny that migrate along the outer root sheath (ORS), enveloping the hair follicle, and express some markers at the base of the hair follicle to generate a specialized, highly proliferative cell population that supports the growth of regenerating follicles (Blanpain and Fuchs, 2009). This intricate system of stem cells and their progeny ensures the continuous renewal and repair of the skin in response to physiological and pathological challenges.

The intricate organization and diversity of markers associated with adult epithelial stem cells in the intestine, lung, and skin, and the detailed characterization of these stem cell populations highlights their specialized roles in maintaining tissue homeostasis and responding to injuries. The shared markers between organs suggest common mechanisms in the regulation of cell fate, and this is an exciting area of research in stem cell biology.

## ECM components

The definite location of these stem cells in their respective organs, such as the intestinal crypts, distal airways, and hair follicles, emphasizes the significance of the microenvironment or niche in which they reside. The extrinsic signals provided by the local microenvironment play a critical role in regulating the responses and fate decisions of stem cells.

The ECM provides physical support and, more importantly, serves as a reservoir for various biochemical and biophysical cues that can influence stem cell behavior, and is known to play a role in regulating stem cell fate decisions, including proliferation, differentiation, and self-renewal. The dynamic interplay between stem cells and their ECM microenvironment contributes to tissue homeostasis and regeneration. When examining ECM components in different organs, the similarity in composition becomes apparent (Trentesaux et al., 2020), encompassing various types of collagens, laminins, fibronectin, and several proteoglycans. Numerous studies have investigated the matrisome of these epithelial tissues, revealing striking similarities (See Table 1).

**TABLE 1:** Summary of stem cells main markers and ECM components of this compartment.

Stem cells	Markers	Glycoprotein	Proteoglycans
Gut	Lgr5, Bmi1, mTert, Hopx,Lrig1	Collagen (I III IV VI) Laminin Fibronectin Tenascin-c Mucin Epimorphin	Syndecan Versican Decorin Aggrecan lumican Biglycan Heparate sulfate
Skin	Lgr5, CD34, K15, Lrig1,Lhx2, Sox9, Col17a1	Collagen (I III IV V VII) Elastin Laminin Fibronectin Tenascin-c Entactine Osteonectin Galectin Nephronectin	Syndecan Versican Decorin Glypican Aggregan
Lung	p63, Krt5, CCSP, SPC, Sca-1	Collagen (I III IV V) Elastin Laminin Fibronectin Tenascin-c Nidogen	Syndecan Versican Decorin Aggregan Heparate sulfate

The intestinal stem cell niche is characterized by a complex network of fibrous structural proteins, including proteoglycans and glycoproteins, that form a scaffold that contributes to the three-dimensional architecture crucial for cellular homeostasis (Meran et al., 2017). The apical part of ISCs is exposed to the intestinal lumen, while their basal part is in direct contact with a matrix component network, constituting the basement membrane, and further connected to the ECM and mesenchymal cells comprising the lamina propria. Collagen types I, III (fibrillar collagen), IV (network-forming), and VI are evenly distributed in the healthy intestinal ECM (Followill and Travis, 1995; Simon-Assmann et al., 1995; Hilska et al., 1998; Handorf et al., 2015). Notably, there is growing evidence that collagen type VI, closely interacting with basement membrane collagen type IV, serves as a key regulator of the mechanical microenvironment of intestinal crypt cells through fibronectin and RGD (Arg-Gly-Asp)-dependent interactions with crypt cells (Groulx et al., 2011; Benoit et al., 2012). Type VI collagen is secreted by the intestinal crypt cells into the basal lamina of the intestinal basement membrane (Groulx et al., 2011). Additionally, intestinal fibronectin, secreted by fibroblasts and expressed by epithelial cells, is distributed throughout the lamina propria (Quaroni et al., 1978; Simon-Assmann et al., 1986). Laminins, a crucial factor for establishing epithelial cell polarity (Simon-Assmann et al., 1994; Teller and Beaulieu, 2001), play a significant role in enhancing ISC survival and proliferation. Finally, glycosaminoglycans (GAGs) represent an important niche for ISC homeostasis.

The lung’s very unique ECM provides structural support for cells and regulates homeostasis and injury-repair responses (Burgstaller et al., 2017). The pulmonary ECM changes significantly during lung development, forming a progressive scaffold for the intricate structure of the lung from the trachea to the alveoli. Distinct ECM components are involved in airway branching and alveolar septation (Zhou et al., 2018). Airway branching requires laminins, fibronectin, tenascin, and syndecan, while alveolar septation involves elastin and tropoelastin, particularly during the saccular stage when airspace expands. To comprehensively address this topic, one must consider the entire matrisome of the adult lung. It has been well described in several inspiring murine studies (Naba et al., 2012; Schiller et al., 2015; Mayorca-Guiliani et al., 2017; Zhou et al., 2018). In summary, it comprises 143 matrisome proteins categorized into two main categories: The core matrisome proteins (glycoproteins, collagens, and proteoglycans) and the matrisome-associated proteins (inclusive of remodeling enzymes and ECM-affiliated proteins).

In more detail, fibrillar collagens, Collagen I and III, are found around airways and blood vessels. Network-forming Collagen IV is located at the basement membrane region of airways and alveoli and around blood vessels (Zhou et al., 2018). The large proteoglycan, Versican, is observed superficially in the airway epithelium, interstitial areas, and immune cells within the alveoli (Hewitt et al., 2023). During early embryonic lung development, all five laminin α chains are present, but normal adult lung tissue primarily contains laminin α3, α4, and α5 chains (Miner et al., 1997). Laminins α1, α2, and α3 are localized in the airway epithelial basement membrane during early lung development (Zhou et al., 2018).

Over the past 30 years, there has been extensive characterization of the expression of ECM proteins and their receptors in the skin (Watt, 2002; Wilhelmsen et al., 2006; Sugawara et al., 2008; Breitkreutz et al., 2009; Ko and Marinkovich, 2010; Dussoyer et al., 2022; Li et al., 2022). Recently, the first description of the skin matrisome in healthy adult mice identified 236 proteins, including 95 core matrisome proteins and 141 associated matrisome proteins. Among the 1,112 components described in the mouse matrisome, 236 were found in the skin of healthy adult mice, constituting 21% of all matrisome content (http://matrisomeproject.mit.edu/) (Naba et al., 2012). Roig-Rosello and others describe that the 95 core matrisome proteins include 30 collagen chains, 53 glycoproteins, and 12 proteoglycans. Among those core components several collagens such as collagen I, III, V, VI, XII, and XIV are found in the dermis, and the collagen IV, VII, XVII, and XVIII in the dermoepidermal junction. Interestingly, collagen XV and XVI are common to both dermis and dermoepidermal junction matrisomes (Ricard-Blum, 2011; Theocharidis and Connelly, 2019; Roig-Rosello and Rousselle, 2020).

Several laminins, proteoglycans, and ECM regulators are also present. Specialized studies have highlighted specific ECM niche components for stem cells, such as Col17A1, Tenascin C, Nephronectin, and Embiggin (Fujiwara et al., 2011; Hendaoui et al., 2014; Chen et al., 2015; Matsumura et al., 2016; Sipilä et al., 2022). However, to integrate these components into a signaling scheme, this inventory of ECM components should be considered in conjunction with the pattern of integrin receptor expression. Elevated levels of integrin expression have long been recognized as a marker of epidermal stem cells (Jones and Watt, 1993; Jones et al., 1995). The crucial role of the nephronectin/α8 integrin ligand-receptor pair has highlighted in Fujiwara H, et al. (Fujiwara et al., 2011). The hair follicle niche, associated with the arrector pili muscle, displays a distinctive enrichment in nephronectin, a Wnt target gene. Nephronectin, a ligand of α8 integrin expressed by bulge stem cells, provides anchorage to arrector pili muscle progenitors, creating a functional niche for hair follicle stem cells (Fujiwara et al., 2011). The activity of stem cells from the hair follicle is highly regulated by ECM components signaling through the Wnt pathway. Both type VI collagen and tenascin C contribute to hair follicle stem cell function through Wnt signaling regulation (Hendaoui et al., 2014), illustrating the link between ECM molecules and signaling pathways through the immobilization of ligands on ECM. Recently, the expression of the transmembrane protein Embigin by sebaceous gland (SG) cells revealed a new ECM-specific pattern that functionalizes a niche. Embigin modulates ECM organization by binding fibronectin and facilitating basolateral targeting of monocarboxylate transport. These results underscore the molecular mechanism coupling adhesion and metabolism regulated by Embigin (Sipilä et al., 2022).

## Mechanosignaling in the three epithelia

The downstream signaling of the ECM, namely mechanosignaling, has been a subject of investigation and initial evidence obtained through cultured cells suggests that epidermal stem cells are regulated by integrin-ECM binding. In a pioneering study in 1988, Watt, Jordan, and O’Neill (Watt, 1988) utilized adhesive micropatterns to fix and organize cell geometry, a biophysical technique still widely used nowadays. Epidermal stem cells on small micropatterns exhibited a rounded shape, reduced DNA synthesis, and increased expression of keratinocyte differentiation genes (Watt, 1988). Conversely, cells on larger micropatterns maintained a low level of differentiation. This established a direct link between the adhesion surface of epidermal stem cells, their shape, and terminal differentiation. It opened new perspectives, suggesting that cells could sense density, leading to the concept of regulation of self-renewal and differentiation by cell size (Watt, 1988).

ECM components form a complex network at the cell surface, recognized by specialized membrane receptors, called integrins. Integrins bind extracellular fibrils and interact with actin microfilaments in the intracellular compartment, providing a direct link between extracellular and intracellular spaces (Schwartz, 2010). Each element of the scaffold, from the cytoskeleton to the integrin-mediated adhesions to the extracellular matrix, transmits forces that can originate from both intracellular contractile forces generated by myosin and forces from outside the cell. These forces activate a wide range of signaling pathways among them YAP/TAZ (see below). Indeed, focal adhesion structures transmit the mechanical forces from large integrin complexes at the plasma membrane to the intracellular cytoskeleton. (Rausch and Hansen, 2020). The increased stiffness is transmitted through integrin ß1 and activates FAK, which in turn activates Src, which subsequently leads to the activation of YAP (Kim and Gumbiner, 2015; Mason et al., 2019).

Yes-associated protein 1 (YAP1) and WW-domain-containing transcription regulator 1 (WWTR1; a.k.a. TAZ) are transcriptional coactivators. Both proteins must interact with DNA-binding factors to modulate transcription. Specifically, TEAD family members are major transcriptional enhancer factors for YAP/TAZ (Dupont et al., 2011). YAP/TAZ activity is characterized by the tightly regulated balance of nuclear to cytoplasmic ratio of unphosphorylated and phosphorylated YAP/TAZ leading to lineage fate determination (Piccolo et al., 2014). YAP/TAZ signaling has been described as crucial regulators of cell fate commitment from embryonic stem cells (ESC) to adult stem cells. YAP silencing leads to loss of murine ESC pluripotency (Tamm et al., 2011). In adult tissues, nuclear YAP and TAZ are commonly located in enriched area for somatic stem cells or progenitors, such as the bottom of intestinal crypts and the basal layer of the epidermis (Schlegelmilch et al., 2011; Barry et al., 2013; Panciera et al., 2017). In skin, YAP/TAZ induce proliferation of epidermal stem cells, when high levels of YAP/TAZ activity promote proliferation and inhibition of differentiation of intestine stem cells (ISC) (Schlegelmilch et al., 2011; Le Bouteiller and Jensen, 2015).

In ICS (Houtekamer et al., 2022), β1-integrins have been identified as important regulators of proliferation and homeostasis by mediating Hedgehog signaling in a genetic study in mice (Jones et al., 2006). Additionally, β1-integrins via an integrin-linked kinase (ILK) -fibronectin-dependent mechanism regulate ISCs fate (Gagné et al., 2010). An elegant study demonstrated that during intestinal repair, the epithelium is transiently reprogrammed into a primitive state. This fate adjustment is driven by ECM remodeling, that leads to an increased FAK/Src signaling, and ultimately YAP/TAZ activation (Yui et al., 2018).

In the lung, AT1 cells are required for gas exchange through the endothelial capillary network, while AT2 cells produce and reprocess pulmonary surfactant and serve as optional progenitors that differentiate into AT1 cells (Barkauskas et al., 2013; Zacharias et al., 2018). The AT1 cell fate is actively regulated by YAP/TAZ, and their loss leads to a rapid reprogramming of AT1 into AT2 cells. The presence of nuclear YAP in AT1 and not AT2 cells suggests a specific responsiveness to Hippo signaling in the AT1 lineage. However, overexpression of a constitutively active YAP protein in AT2 cells can lead to increased expression of some AT1 cell markers. Even though, these cells do not display all traits of this lineage, it does include a consistently enlarged squamous shape. Penkala et al. elegantly describe mechanotransduction as a key actor in AT1 rather than AT2 cells to maintain alveolar function in the lung (Penkala et al., 2021).

Physical stress, such as stretching, promotes proliferation (Jones et al., 2006), metabolism of prosurfactant, cellular damage or death (Arold et al., 2009), and migration (Desai et al., 2008) of AT2 cells. Alveolar epithelial cells mitotic activity, growth factor production are regulated by mechanical forces. Physical strain also regulates the expression of platelet-derived growth factor receptor beta (PDGFRB) during lung development. AT2 cells cultured with conditioned media from lung fibroblasts were subjected to mechanical stretch cycle that leads to increased DNA synthesis compared to non-mechanically stimulated ones (Noskovičová et al., 2015). AT2 main function is to produce and secrete pulmonary surfactant. Cyclic stretch increases secretion of surfactant by increasing rapidly intracellular calcium levels, thus reinforcing AT2 specific function (Asmani et al., 2019).

Recently, a new study established that mechanical properties define AT1 cells *in vivo* and that the AT1 and AT2 cell fate transitions are tuned through mechanism involving actin dynamics and integrin signaling (Shiraishi et al., 2023). AT1 cell identity is dependent on physical respiratory movement. Using lamin B1 ChIPSeq, the authors analysed genome organization under breathing constraints in AT1 and AT2 cells. They specifically studied Lamina-associated domains (LADs). LADs are genomic elements of transcriptionally repressed chromatin located at the nuclear periphery. LADs influence 3D genome architecture by their association with the nuclear lamina and subsequent interactions with the multiprotein LInker of Nucleoskeleton and Cytoskeleton (LINC) complex (Briand and Collas, 2020). Bioinformatics Ingenuity Pathways Analysis (IPA) revealed that AT2 LAD genes were enriched for genes linked to actin cytoskeleton regulation and focal adhesion, thus repressed by spatial repositioning to the nuclear periphery. Based on this data, a working model was described where the nuclear-lamina interactions play a central role in discriminating AT1 and AT2 cell fate. Overall, the authors clearly demonstrated that alveolar cell fate is dependent on active breathing movements (Shiraishi et al., 2023).

Several studies on the skin have described the fundamental role of mechanosignaling in regulating epidermal fate. Indeed, in the basal layer of the epidermis, direct interaction with the basement membrane leads to Integrin/src signaling activation that constitutes the crucial step in the nuclear translocation of YAP/TAZ (Elbediwy et al., 2016). Moreover, Sebaceous Glands expand within a compliant microenvironment during morphogenesis, and changes in physical properties from the structural surroundings will influence global tissue homeostasis (Andersen et al., 2019). Quantification and modeling of tissue physical deformation in the epidermis lead to the description of this balance regulation through the integration of locally applied mechanical forces coming from both cell proliferation and desquamation. This study reveals how singular behaviors are integrated into a biochemical signaling hub linking proliferation, cell fate and location to build a stratified epithelium (Miroshnikova et al., 2018). Another study demonstrates that the integrin coreceptor and amino acid transporter SLC3A2 regulate skin homeostasis by controlling the sphingolipid metabolic pathway, which, in turn, modulates the perception of integrin stiffness. SLC3A2 increases sphingolipid availability, thus supporting proper membrane recruitment and shuttling of upstream RhoA regulators and GEFH1. This intricated regulation between integrin mechanosensing and cellular metabolism will give a critical framework that participates to cutaneous mechanical homeostasis (Boulter et al., 2018).

Among numerous signaling processes that regulate SC fate, epigenetics has emerged as a crucial feature. Epigenetic modifications are heritable changes that affect gene expression but they do not involve changes in the DNA sequence (Goldberg et al., 2007). They include but are not restricted to DNA methylation and histone modifications. The landscape defined by those modifications plays key role in the regulation of stem cell fate (Teschendorff and Feinberg, 2021). For example, the polycomb PRC2 complex containing Ezh2 maintains ISC population by promoting proliferation and repressing lineage differentiation choices (Chiacchiera et al., 2016; Koppens et al., 2016). Another example in the epidermis, the polycomb complex including Ezh2 tunes the cell fate choice of HFSCs (Ezhkova et al., 2009). In the bulge of the hair follicle, Ezh2 mRNA is downregulated during hair follicle regression, whereas Ezh1 remains expressed. Intriguingly, a small subset of genes acquires H3K27me3 marks at the same stage, as shown by Chip-Seq analysis. Ezh1 and Ezh2 seem to play differential role that cannot be completely redundant (Lee et al., 2016).

Polycomb complexes maintain the stem cell proliferative potential and globally repress unwanted differentiation programs while selectively establishing a specific terminal differentiation program in a finely tune manner.

Interestingly, mechanotransduction has recently been shown to influence epigenetic traits by acting directly on nuclear shape or by directly integrating mechanosensitive signals. (Kalukula et al., 2022). Several inspiring studies have described the role that ECM stiffness plays in modulating epigenetics, either through DNA methylation or histone modifications in the control of stem cell differentiation (Song et al., 2020).

In skin, epidermal stem cells express a mechanosensory complex of emerin (Emd), non-muscle myosin IIA (NMIIA) and actin that controls gene silencing and chromatin compaction. When subjected to force, Emd is enriched at a specific site on the outer nuclear membrane, leading to a lack of heterochromatin anchoring. This defective anchoring triggers a switch in histone methylation from H3K9me2,3 to H3K27me3. It also increases actin polymerization, leading to a lack of nuclear actin which in turn results in an accumulation of H3K27 me3 marks at specific sites of heterochromatin. This establishes a link between the biophysical properties of the niche and the changes in the nucleus, thus controlling lineage commitment. (Le et al., 2016).

## What about aging?

Considering all these studies, it becomes evident that YAP/TAZ-mediated mechanosignaling is a key regulator of cell fate in adult epithelia, independent of their intrinsic proliferation status. It should not be forgotten that mechanosignaling in the epithelium is not limited to integrin-dependent signals, but also occurs via adherens junction and desmosomes, which gives an even more sophisticated picture (Rübsam et al., 2018). This leads to the question of whether the source of longevity could be concealed in the scaffold provided by the specific ECM composition surrounding resident stem cells. One remarkable hallmark of aging is the modification of ECM composition, leading to changes in the biophysical attributes of the niche. It has been extensively described that aging affects the assembly and stiffness of the environment. The intestine and intestinal stem cells (ISCs) exhibit decreased renewal abilities with aging (Martin et al., 1998; Nalapareddy et al., 2017; 2022; Pentinmikko and Katajisto, 2020). Recent studies in the field report these decrease capacities and link them to both cell-autonomous processes (Martin et al., 1998) and non-cell-autonomous defects, such as the depletion of niche-derived Wnt signals (Nalapareddy et al., 2022).

Changes in the ECM impact both the lower airway and alveolar spaces, affecting progenitor cell function. Quantitative and qualitative changes through age have been documented for some lung progenitor populations. They display different behavior: Basal and club cells decrease in number with age, whereas AT2 cells number remain unchanged but harbor decreased functionality such as self-renewal and differentiation capacity (Wansleeben et al., 2014; Ortega-Martínez et al., 2016; Han et al., 2023). Moreover, using alveolar organoids, it has been shown that isolated AT2 cells from old mice display a reduced capacity to form alveolar organoids when compared to young adult mice (Rowbotham et al., 2023). The combination of both cell-autonomous and non-cell-autonomous mechanisms powers these age-related phenotypes. In polyploidy, cells have a mechanism to become larger as DNA content scales with cell size, which is a purely cell-autonomous mechanism of progenitor cell dysfunction (Ganem and Pellman, 2007; Selmecki et al., 2015). In the skin, aging induces a reduced capacity of epidermal stem cells to maintain hair follicle homeostasis and wound repair. The proteolysis of Col 17 A1, a main hemidesmosomal component and stem cell marker, occurs during aging as a consequence of DNA damage accumulation over time, leading to hair follicle miniaturization and depletion of hair follicle stem cells (HFSCs) (Matsumura et al., 2016). Additionally, altered ECM gene expression has been described during defects in hair *de novo* formation following a wound. Interestingly, aged epidermal stem cells capacity can be restored if associated to neonatal dermis in functional grafting assays (Ge et al., 2020; Raja et al., 2022). Furthermore, aged HFSCs display an extensive reduction in chromatin accessibility. This occurs specifically at crucial self-renewal and differentiation genes that are regulated by bivalent promoters. These promoters present both active and repressive marks (Koester et al., 2021). Mechanistically, the aged HFSC niche exhibited extensive changes in ECM components and biophysical properties, leading to mechanical defects and concomitant transcriptional repression to shut down promoters (Koester et al., 2021).

If these three epithelia share common features and homeostasis mechanisms, aging, as a normal physiological challenge, is indeed included in the common characteristics of the three epithelia. We apologize to the numerous authors who have largely contributed to this growing field and whom we omitted to cite. Thanks to all the efforts of the scientific community, we now have a clearer idea of the implication of ECM cues during cell fate decisions throughout life. By comparing these three epithelia side by side, we see that the apparent discrepancies in terms of proliferation, repair, and regenerative capacity could be reconciled by considering the environmental cues received by the cell from the niche as the heart of the concept of the scaffold of destiny. Indeed, the ECM scaffold is required to build a tissue dynamic enough to maintain and react to external challenges, ensuring the maintenance of a very fit equilibrium. Understanding the commonalities and discrepancies in the processes that regulate stem cell fate across different organs can provide valuable insights for regenerative medicine and tissue engineering. Harnessing the cues provided by the ECM and deciphering the signaling pathways involved in these processes could result into novel therapeutic strategies for tissue repair and regeneration. The interdisciplinary approach, combining cell biology, molecular biology, and biomechanics, is critical for unravelling the complexities of stem cell regulation in various tissues.

## Abbreviations

ECM, Extra Cellular Matrix; ISCs, Intestinal Stem Cells; CBC cells, Crypt Base Columnar cells; BADJs, bronchioalveolar duct junctions; AT1, Alveolar cells Type 1; AT2, Alveolar cells Type 2; BASCs, bronchoalveolar stem cells; ORS, outer root sheath; SG, sebaceous Gland; YAP, (Yes-associated protein); TAZ, (transcriptional coactivator with PDZ-binding motif); HFSCs, Hair Follicle Stem Cells.
